# Low Cost Embedded Copper Mesh Based on Cracked Template for Highly Durability Transparent EMI Shielding Films

**DOI:** 10.3390/ma15041449

**Published:** 2022-02-15

**Authors:** Anton S. Voronin, Yurii V. Fadeev, Mstislav O. Makeev, Pavel A. Mikhalev, Alexey S. Osipkov, Alexander S. Provatorov, Dmitriy S. Ryzhenko, Gleb Y. Yurkov, Mikhail M. Simunin, Darina V. Karpova, Anna V. Lukyanenko, Dieter Kokh, Dashi D. Bainov, Igor A. Tambasov, Sergey V. Nedelin, Nikita A. Zolotovsky, Stanislav V. Khartov

**Affiliations:** 1 Department of Molecular Electronics, Federal Research Center «Krasnoyarsk Scientific Center», Siberian Branch, Russian Academy of Sciences (FRC KSC SB RAS), 660036 Krasnoyarsk, Russia; daf.hf@list.ru (Y.V.F.); michanel@mail.ru (M.M.S.); dankerik@mail.ru (D.V.K.); diter.koh@gmail.com (D.K.); stas_f1@list.ru (S.V.K.); 2 School of Engineering and Construction, Siberian Federal University, 660041 Krasnoyarsk, Russia; 3 Laboratory of EMI Shielding Materials, Bauman Moscow State Technical University, 105005 Moscow, Russia; m.makeev@bmstu.ru (M.O.M.); pamikhalev@bmstu.ru (P.A.M.); osipkov@bmstu.ru (A.S.O.); htet2066@gmail.com (A.S.P.); dsr@bmstu.ru (D.S.R.); ygy76@mail.ru (G.Y.Y.); 4 Laboratory of Reinforced Plastics, N.N. Semenov Federal Research Center of Chemical Physics, Russian Academy of Sciences, 119991 Moscow, Russia; 5 School of Non-Ferrous Metals and Materials Science, Siberian Federal University, 660041 Krasnoyarsk, Russia; 6 Department of Aircraft, Reshetnev Siberian University Science and Technology, 660037 Krasnoyarsk, Russia; 7 School of Engineering Physics and Radio Electronics, Siberian Federal University, 660041 Krasnoyarsk, Russia; lav@iph.krasn.ru (A.V.L.); s.v.nedelin@mail.ru (S.V.N.); nikitazolotovskiy@mail.ru (N.A.Z.); 8 Laboratory of Radiospectroscopy and Spintronics, L.V. Kirensky Institute of Physics, Siberian Branch, Russian Academy of Sciences, 660036 Krasnoyarsk, Russia; 9 Scientific and Training Center of Space Research and High Technologies Institute, Reshetnev Siberian University Science and Technology, 660037 Krasnoyarsk, Russia; 10 Laboratory for Radiation and Plasma Technologies, Tomsk Polytechnic University, 634050 Tomsk, Russia; das@tpu.ru; 11 Laboratory of Radiophotonics, V.E. Zuev Institute of Atmospheric Optics, Siberian Branch, Russian Academy of Science, 634055 Tomsk, Russia; 12 Laboratory of Photonics of Molecular Systems, L.V. Kirensky Institute of Physics, Siberian Branch, Russian Academy of Sciences, 660036 Krasnoyarsk, Russia; tambasov_igor@mail.ru; 13 LLC Research and Production Company “Spectehnauka”, 660043 Krasnoyarsk, Russia

**Keywords:** transparent electromagnetic interference (EMI) shielding films, cracked template, electroplating, photocurable resin, embedded mesh, durability

## Abstract

Embedded copper mesh coatings with low sheet resistance and high transparency were formed using a low-cost Cu seed mesh obtained with a magnetron sputtering on a cracked template, and subsequent operations electroplating and embedding in a photocurable resin layer. The influence of the mesh size on the optoelectric characteristics and the electromagnetic shielding efficiency in a wide frequency range is considered. In optimizing the coating properties, a shielding efficiency of 49.38 dB at a frequency of 1 GHz, with integral optical transparency in the visible range of 84.3%, was obtained. Embedded Cu meshes have been shown to be highly bending stable and have excellent adhesion strength. The combination of properties and economic costs for the formation of coatings indicates their high prospects for practical use in shielding transparent objects, such as windows and computer monitors.

## 1. Introduction

The space around us is permeated with electromagnetic radiation of various frequencies. In the range from tens of kHz to hundreds of GHz lie such essential technologies as radio navigation, mobile and satellite communications, TV and radio broadcasting, Wi-Fi, etc. For the correct operation of high-precision electronic devices, human life support systems, as well as information protection, it is crucial to effectively protect such devices from parasitic radio emission, which is implemented mainly through the use of materials, design and technological solutions based on them providing radio shielding in various frequency ranges. At the same time, at the moment, there is an increasing demand for materials that, in addition to shielding, have a different combination of such performance properties as flexibility, transparency in various spectral ranges, low weight and thickness, air permeability, etc.

To date, significant progress has been made in developing shielding materials. In particular, such lightweight, durable and affordable materials as carbon nanotubes, graphene, MXene paper [1,2,3,4], aerogels [5,6,7,8] and polymer composites [9,10] and copper nanosheets films [11] with a shielding coefficient *SE* not less than 60–100 dB have been developed. At the same time, these materials are opaque in the visible range. They are not suitable for solving problems of shielding transparent objects: displays of smartphones, computers and measuring instruments, glazing elements of special-purpose buildings, etc.

The best balance between optical transparency and shielding is metallic micro and nanostructures. In the context of transparent, high-performance coatings with *SE* more than 30 dB, it is advisable to consider layers based on highly conductive metals, for example, Ag nanowires [12,13,14,15] and Cu nanowires [16]. Additional electroless deposition of Cu on Ag nanowire seed contributes to a dramatic increase in *SE* for a wide frequency range [17]. Lithographic micro and nanomeshes, also widely presented in the literature, demonstrate high optoelectric parameters and *SE*: Ag ring micromesh [18], Cu square nanomesh [19], Cu-Ni square micromesh [20] and Ag-Ni stochastic micromesh [21]. Recently, much attention has been paid to obtaining micro and nanomesh coatings based on self-organization processes. Such methods can significantly reduce the cost of coating production and achieve high optoelectric parameters and *SE*. The following solutions can be cited as example: cracked template Ag mesh [22] and Cu-Ag; Ni-Ag meshes [23]; and Ag nanoparticle mesh emulsion template [24]. An important feature of the cracked template is the non-periodic mesh structure; therefore, in a multilayer version, the system will not have a moiré pattern [21,23], which is typical for the imposition of periodic meshes [19].

**Figure 1:**
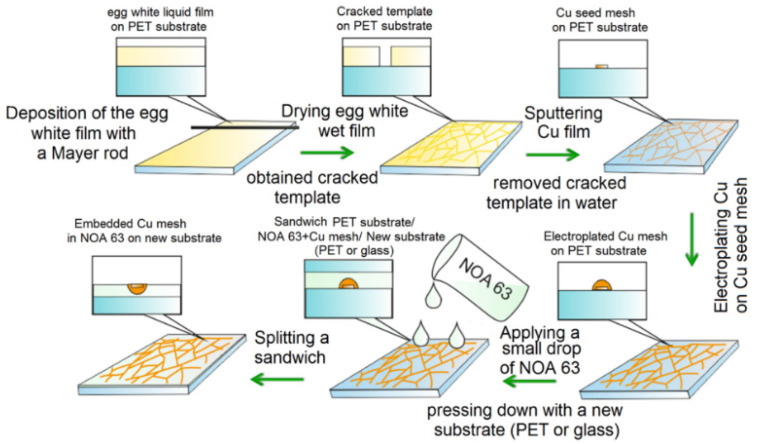
Technological process for obtaining an embedded Cu mesh transparent conductive coating.

This paper proposes a simple strategy for producing embedded Cu meshes obtained by electroplating copper on a Cu seed mesh formed based on a cracked template, followed by embedding in a photocurable resin (Figure 1).

From an economic point of view, Cu seed mesh is more profitable than Ag seed mesh, which we used in [23]. The technological process of embedding a mesh into a photocurable resin layer contributes to a significant increase in the mechanical adhesion properties of the coating.

We believe that in solving the problem of shielding transparent objects, these materials will become an excellent alternative to micro and nanomeshes obtained by lithography methods, since these methods significantly limit the possibility of scaling the technology over large areas and increase the cost of such materials.

## 2. Materials and Methods

### 2.1. Materials

Egg white, egg yolk obtained from a food market. Copper sulphate pentahydrate (CuSO_4_·5H_2_O), sulfuric acid (H_2_SO_4_), ethanol (C_2_H_5_OH), all pure chemical reagents. Cu target (99.99%) (Kurt J. Lesker Company, Jefferson Hills, PA, USA). Transparent photocurable resin NOA 63 (Norland Products Inc., Jamesburg, NJ, USA). PET substrates 50 µm thick (Hi-Fi Industrial Film Ltd., Stevenage, Hertfordshire, UK).

### 2.2. Cracked Template Preparation

Our previous work [23] describes the method for preparing an egg white precursor in detail. The egg white precursor for the cracked template was prepared according to the following procedure: in a fresh chicken egg, the white was separated from the yolk, then the white was thrown onto a coarse sieve with a mesh period of 500 µm. On a sieve, the egg white was separated into two fractions; the fluid mobile fraction flowed into the glass, and the viscous jelly-like fraction remained on the sieve. For the experiments, we used a fluid fraction of egg white, to which we added egg yolk. Egg white solutions with a yolk concentration of 0.1 g/L (hereinafter, mesh coating based on this template will be called Large Cell Mesh or LCM) and 3 g/L (hereinafter, mesh coating based on this template will be called Small Cell Mesh or SCM).

**Figure 2:**
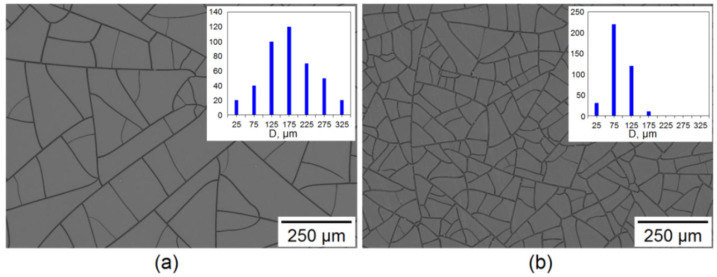
SEM image of cracked templates for LCM (**a**) and SCM (**b**); histograms of average cell size distribution shown in insets.

The paper considers two types of templates to obtain mesh coating with different Fill Factors (*FF*). Egg white solutions were applied by the Mayer rod method onto PET substrates 50 µm thick. The thickness of the egg white layer was 50.8 µm (Mayer rod # 20), and the deposition rate was 30 mm/s. After application, the liquid film was dried at room temperature 20 °C. SEM image of cracked templates is shown in Figure 2.

According to statistical analysis, the LCM template has an average cell size of 177.5 ± 72.9 µm (Figure 2a inset), and the SCM template has an average cell size of 76.5 ± 35.5 µm (Figure 2b inset). Thus, we consider a cracked template with an almost twofold difference in the average cell size in our work.

### 2.3. Magnetron Sputtering of Cu Layer and Obtained Cu Seed Mesh

The deposition of the Cu layer on the templates was carried out by magnetron sputtering of a copper (99.9% purity) rectangular target with a size of 700 × 100 mm^2^. Argon with a purity of 99.99% was used as a working gas. Argon was supplied to the working chamber through a mass flow controller. The working chamber was evacuated using a turbomolecular pump. The residual and working gas pressures were 3.0 × 10^−3^ Pa and 4.0 × 10^−1^ Pa, respectively. The templates were placed at a distance of 10 cm from the target. Power to the magnetron sputtering system was supplied from a unit generating DC pulses with a frequency of 133 kHz. Spraying took place in the mode of stabilization of the discharge power, which was maintained at a level of 1.5 kW. In this case, the discharge current was 2.7 A.

A quartz sensor was used to control the Cu film thickness by measuring the resonant frequency change. The LCM was sputtered with 300 nm Cu, and the SCM was sputtered with 100 nm Cu. The reason for the different thicknesses of the seed mesh structure is due to the fact that for the LCM template, due to the small fill factor, the surface resistance of the seed mesh with a thickness of 100 nm was more than 150 Ω/sq. It has been observed that the high resistance of the seed mesh leads to non-uniform growth of electroplated copper, which is unacceptable for the proposed process. Therefore, we deliberately increased the thickness of the seed Cu mesh for the LCM template to 300 nm.

### 2.4. Electroplating Copper on Cu Seed Mesh

Electroplating of Cu was carried out in a potentiostatic mode; the operating voltage was 0.5 V. The mass transfer of metals to the seed Cu meshes was controlled by the time of the process; in this work, deposition for 30 s and 180 s was chosen. The parameters of the copper plating electrolyte are described in detail in [23]. Further in the text, we will use two types of designations—markings of LCM 0, LCM 30 and LCM 180 mean that this is Cu seed mesh and electroplated meshes with a settling time of 30 and 180 s based on a template with large cell size (Figure 2a). Correspondingly, SCM 0, SCM 30 and SCM 180 are the same for the small cell size template (Figure 2b).

### 2.5. Embedding Process Cu Meshes in Photocurable Resin Layer

The embedded mesh formation technique was as follows: 0.1 mL of NOA 63 was applied to a Cu mesh on PET (2.5 × 3 cm^2^) at the first stage. From above, the drop was smoothed using another substrate, which became the carrier for the embedded Cu mesh; either PET or glass was used. The resulting sandwich was pressed for 1 min with a force of 100 N. After pressing, without relieving the pressure, the operation was exposed to UV radiation (20 W, λ = 365 nm) for 10 min. During that time, the photocurable resin is completely cured and reliably retains elecroplated Cu mesh. After the embedded operation, the labelling of the samples is changed, and e-(i.e., embedded) is added to the sample name. Thus, embedded Cu meshes will be designated in the text, for example, e-LCM 180 and e-SCM 180.

### 2.6. Morphology Study Cu Meshes and Embedded Cu Meshes

The morphology of the cracked templates and different mesh types was studied by scanning electron microscopy using a TM-4000 Plus (Hitachi, Tokyo, Japan), accelerating voltage 15 kV, and SEM equipment energy-dispersive X-ray spectrometer X-Flash 630Hc (EDS, Bruker, Billerica, MA, USA).

The local surface morphology and thickness of meshes were determined by atomic force microscopy (AFM), DPN 5000 NanoInk (Nanoink Inc., Skokie, IL, USA). For best results, scanning was carried out in semi-contact mode using high-resolution cantilevers VIT_P/IR (TipsNano, NT-MDT Spectrum Instruments, Zelenograd, Moscow, Russia) with a curvature radius of 10 nm.

### 2.7. Optoelectrical Properties Embedded Cu Meshes

The spectral dependencies of the optical transmittance of the meshes were measured in the range of 400–700 nm with a spectrophotometer UV-3600 (Shimadzu, Kyoto, Japan).

A four-probe method was used for sheet resistance measurements using a Keithley 2000 multimeter (Keithley Instruments, Solon, OH, USA) and a four-probe head Mill-Max 854-22-004-10-001101 (Mill-Max Mfg. Corp., Oyster Bay, NY, USA).

### 2.8. Mechanical Properties of Cu Meshes and Embedded Cu Meshes

The adhesion strength of Cu meshes and embedded Cu meshes was evaluated using a tape test according to ASTM D 3359, in geometry B (notch lattice period was 2 mm). The effect of the reusable tape test on sheet resistance Cu meshes and embedded Cu meshes was also studied. 3M tape was used in all studies, and the test sample was firmly fixed on the table.

The mechanical properties of mesh coatings were studied in comparative experiments at a laboratory stand in a cyclic mode [23]. Cyclic bending allows exploration of the accumulation of fatigue in meshes. In our experiments, the number of bending cycles with a radius of 5 mm was 1000.

### 2.9. Shielding Properties of Embedded Cu Meshes

In this study, the *SE* of the materials was measured using a special 16.00/6.95 mm air-filled coaxial cell (type II, 50 Ω, GOST RV 51914-2002). The advantages of this approach are a wide frequency range (from 10 MHz to 7 GHz), the ability to measure at low frequencies and a relatively simple and convenient measurement technique. It is required to ensure reliable electrical contact between the material sample and the conductors of the coaxial path over the entire area of their contact for the correctness of the result. To overcome this difficulty, the specific design of the cell has a groove of varying thickness into which the sample is inserted and clamped. Electro-sealing gaskets made of dense metallized fabric were used for better electrical contact between the sample and the coaxial tract walls. The cell provides the correct result for conductive samples.

The test bench includes a Keysight FieldFox N9916A (Keysight Technologies, Santa Rosa, CA, USA) network analyzer, coaxial cable assemblies and a cell; we performed a full two-port calibration at the cell inputs. To determine the *SE* of a material sample:

1. measure the spectrum *S*_21_ (or *S*_12_) of the cell without a sample (groove thickness— 0 mm), S_21*OS*_ (dB),
2. measure the spectrum *S*_21_ (or *S*_12_) of the sample cell, S_21*S*_ (dB),
3. calculate the spectrum *SE* (dB) by Formula (1),

(1) $$SE=S_{21OS}-S_{21S}$$

The dynamic measurement range of the *SE* unit is 80 dB and the measurement error is ±2 dB.

## 3. Results and Discussion

### 3.1. Morphology Study of Cu Meshes

**Figure 3:**
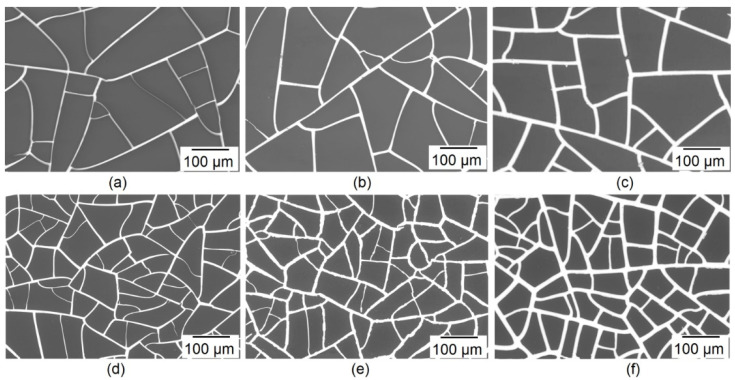
SEM image of Cu meshes before embedding in photocurable resin LCM 0 (**a**); LCM 30 (**b**); LCM 180 (**c**); SCM 0 (**d**); SCM 30 (**e**); SCM 180 (**f**).

The morphology and geometric characteristics of LCM and SCM are the defining parameters that have a key influence on the optoelectric properties of Cu meshes. Figure 3 shows SEM images of LCM and SCM at X200 magnification for the entire range of electroplating, before the process of embedding in a photocurable resin.

As seen from the SEM images, electroplating leads to a width paths increase and, as a result, to the rise in the *FF*. SEM images show uniform copper deposition on two types of seed meshes.

**Figure 4:**
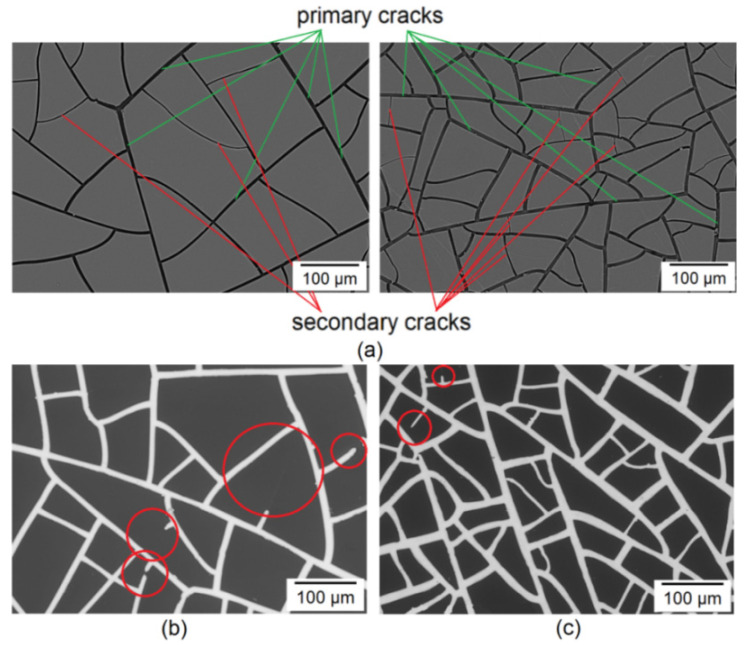
SEM image cracked template LCM (left) and SCM (right) showing primary (green lines) and secondary (red lines) cracks (**a**); Defects on LCM 180 (**b**) and SCM 180 (**c**) (defects indicated by red circles).

The critical question is—What is the defectiveness of the resulting mesh structures? To answer this question, one needs to understand the morphological features of the cracked template. As practice shows, the morphology of a cracked template is formed in several stages [25]. At the first stage, a primary network of cracks (Figure 4a green lines) is formed by relaxing mechanical stresses in the gel layer during its shrinkage. At the second and subsequent stages, in drying out the cracked template, secondary ones are formed (Figure 4a red lines).

Defects arising on electroplated Cu meshes are usually associated with a violation of the integrity of the seed mesh. Based on the cracked template morphology features, it can be seen that secondary cracks have a significantly smaller width than primary cracks. As a rule, the width of secondary cracks does not exceed 1 µm. Narrow paths are easily damaged, resulting in defects in the form of interrupted paths being observed on the surfaces. It is worth noting that the defectiveness associated with undergrowth of lanes, attributable to secondary fractures, appears to be higher in the LCM (Figure 4b) than in the SCM (Figure 4c). This regularity can be explained by the fact that for templates with small cell sizes, the crack length turns out to be shorter, and, therefore, the probability of the appearance of a defect is reduced.

**Figure 5:**
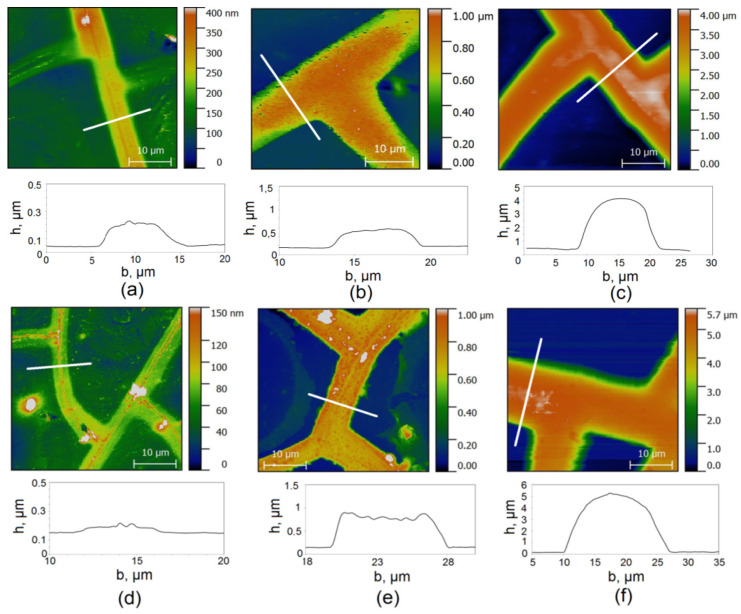
AFM image and profile LCM 0 (**a**); LCM 30 (**b**); LCM 180 (**c**); SCM 0 (**d**); SCM 30 (**e**); SCM 180 (**f**).

Figure 5 shows AFM images for LCM and SCM with different copper deposition times.

AFM data make it possible to objectively estimate the average width of copper paths and their average height for coatings with different electroplating times. Table 1 shows the averaged data for the thickness of the meshes, or the width of the paths.

The *FF* can be estimated according to the following Formula (2).

(2) $$FF=1-\frac{{\left(p-b\right)}^{2}}{p^{2}}$$

where *p* is the average cell size and *b* is the average crack width. Thus, Table 1 provides a complete set of data on the change in Cu meshes’ geometric and morphological characteristics in the course of electroplating.

### 3.2. Morphology Study of Embedded Cu Meshes

The idea of embedding metallic micro and nanostructures into a polymer has two directions: thermal pressing into a thermoplastic polymer and applying the polymer solution to the substrate with its subsequent curing (using a hardener, drying or UV radiation). This strategy makes it possible to significantly increase the physical and mechanical parameters of the coating, such as: adhesive strength, resistance to abrasion and tensile-compression deformation. These approaches are implemented for micromeshes [26,27,28], nanomeshes [29,30], and nanowire films [31,32,33].

**Figure 6:**
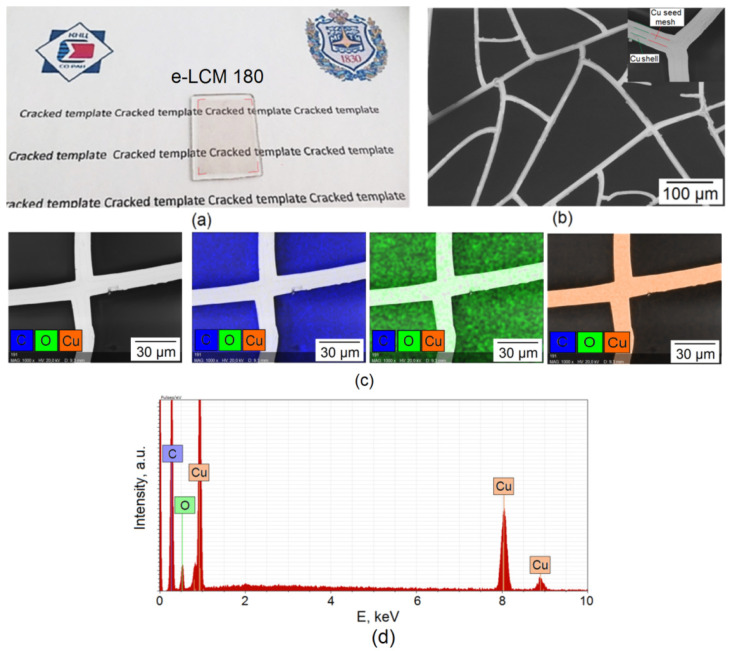
Photo (**a**) and SEM image (**b**); Elemental map (**c**) and EDS spectra (**d**) of e-LCM 180 on glass.

The mechanical properties of the coatings proposed in this work are essential, since they determine their durability and application. Figure 6 shows the appearance (Figure 6a) and SEM image (Figure 6b) of the e-LCM 180 coating on the glass.

SEM analysis of Cu seed mesh interface show that electroplated Cu mesh is of high structural quality (inset Figure 5b). We do not observe cavities and other defects typical for electroplated coatings in the image. Defects are observed on the Cu seed mesh associated with residual protein at the bottom of the cracked template [23]. The high quality of the interface indicates that the egg white adsorbed on the Cu seed mesh during the washing of the template does not significantly affect the quality of the electroplated copper.

EDS analysis e-LCM 180 (Figure 6c,d) demonstrates the presence of three elements: C (*K_α_*_1_ ≈ 0.277 keV), O (*K_α_*_1_ ≈ 0.525 keV) and Cu (*L_α_*_1_ ≈ 0.928 keV and *K_α_*_1_ ≈ 8.046 keV). The distribution of elements exactly corresponds to the mesh structure of the sample (Figure 6c).

**Figure 7:**
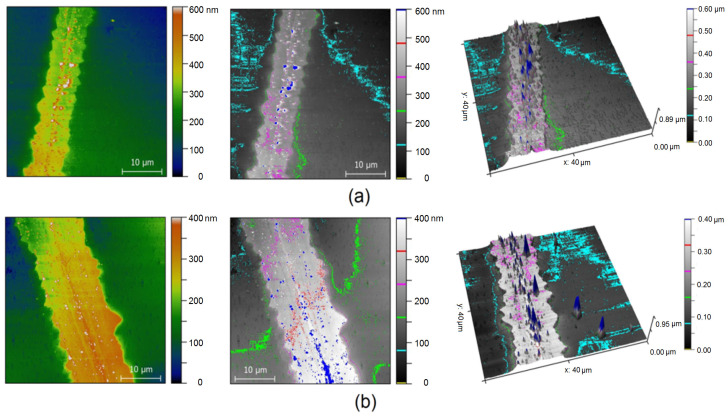
AFM image of e-LCM 30 (**a**) and e-LCM 180 (**b**).

The roughness of embedded meshes was studied using the AFM method. Figure 7 shows embedded meshes e-LCM 30 (Figure 7a) and e-LCM 180 (Figure 7b).

According to AFM, LCM and SCM have significantly lower mesh element heights after embedding. The height of the recessed Cu paths is 100–200 nm, which is well demonstrated by the results of the AFM topography study (Figure 7). Non-zero roughness is due to the excellent adhesion of the Cu seed mesh to the substrate. The greater the thickness of the electroplated Cu, the easier the process of mesh insertion into the photocurable resin carrier.

### 3.3. Optoelectrical Properties of Embedded Cu Meshes

**Figure 8:**
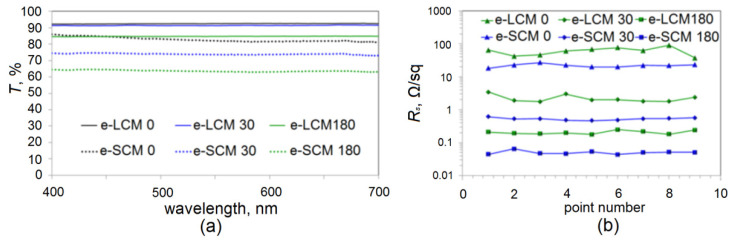
Optical transparency (**a**); sheet resistance measured over the entire area of a 2.5 × 3 cm^2^ sample (**b**).

The analysis of the optoelectric parameters of micro and nanomeshes is quite simple. The analysis of the spectral transmission for meshes with path sizes much larger than the wavelength is reduced to the fact that the mesh elements create a shadow. As a result, the transmission of the coating is proportional to the metallization area. According to [31], the transmission of a metal mesh can be expressed by the formula

(3) $$T=1-FF=\frac{{\left(p-b\right)}^{2}}{p^{2}}$$

where *p* is the period of the mesh and *b* is the path width. Figure 8a shows the spectral dependences for e-LCM and e-SCM coatings with different times of copper electroplating.

In the case of e-LCM 0, there is an integral optical transmission in the considered range of ~92.5%. The deposition of copper for 30 s does not lead to a significant decrease in transparency, which is 91.2%, while sheet resistance (Figure 8b, below we operate with averaged values) decreases significantly from 61.8 Ω/sq to 2.43 Ω/sq. Electroplating Cu for 180 s decreases the integral transparency to 84.3%; however, sheet resistance also decreases to 0.21 Ω/sq. For e-SCM 0, the integral transmittance is lower due to the higher duty cycle and is ~81.9% at a sheet resistance of 21.53 Ω/sq. Due to the small cell size, even 30 s of electroplating reduces the integral transparency to ~73.8%; the sheet resistance was 0.53 Ω/sq. Copper deposition for 180 s showed a sheet resistance of 0.049 Ω/sq with transparency of ~63.2%.

The dependence of the uniformity of sheet resistance on the average cell size is evident (Figure 8b). For e-LCM 0, 30 and 180, the maximum spread of sheet resistance from the average is 45.4, 41.1 and 19.4%, respectively. For e-SCM 0, 30 and 180, the maximum spread from the average is 24.3, 13.1 and 12.2%, respectively.

It can be seen that electroplating reduces the spread of values for both types of coatings. For e-LCM, the specific number of microconductors per unit area can vary significantly due to the large cell size, which is the main reason for the significant error in sheet resistance values. Reducing the average cell size reduces this dispersion, as shown in Figure 8b.

The values of optical transparency and sheet resistance for a transparent conductive coating of any nature can be linked through the function [31].

(4) $$T={\left(1+\frac{Z_{0}}{2R_{s}}\frac{\sigma_{opt}}{\sigma_{dc}}\right)}^{-2}$$

where *T* is the optical transmission at a wavelength of 550 nm; *R_s_*—specific sheet resistance; *Z*_0_ = 377 Ω—vacuum impedance; and the ratio *σ_dc_/σ_opt_* is the main integral characteristic of a transparent electrode [31]. Its value is directly related to the balance between optical transparency and sheet resistance. Thus, the higher the *σ_dc_/σ_opt_* value, the lower the sheet resistance the coating will have at the same transparency. Table 2 shows comparative data of optoelectric characteristics for various transparent conductive layers.

Based on the data in Table 2, it can be concluded that in terms of optoelectric characteristics, e-LCM 30 and e-LCM 180 coatings have great potential in transparent EMI shielding and other important applications, for example, transparent heaters, transparent electrodes for LEDs or OLEDs, photovoltaic cells, etc.

### 3.4. Mechanical Properties of Cu Meshes and Embedded Cu Meshes

The main feature of embedded meshes is their excellent physical and mechanical parameters, such as resistance to bending and abrasion and adhesive strength.

**Figure 9:**
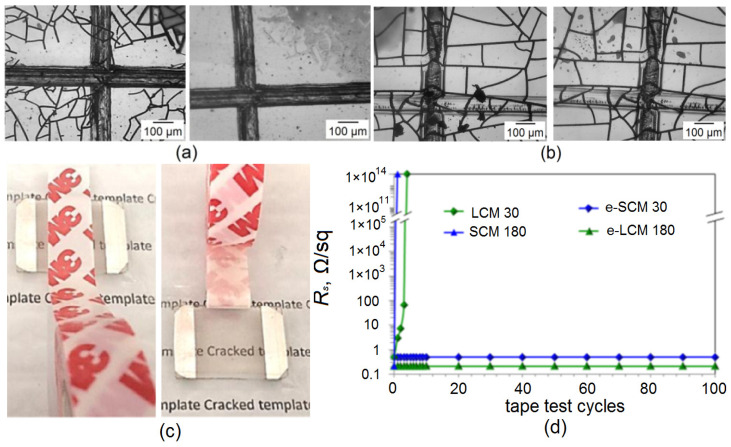
Tape test in B geometry before and after testing SCM 180 (**a**) and e-LCM 180 (**b**); Photo (**c**) and results (**d**) of the cyclic tape test.

We studied the adhesive properties using two approaches. The first approach is a single test to determine the adhesive strength class of the coating according to ASTM D3359. Figure 9 shows microscopic cross-sectional images before (left) and after (right) tape test for SCM 180 (Figure 9a) and e-LCM 180 (Figure 9b).

Figure 9a,b show that PET and NOA 63 have different hardnesses, and therefore the notch mesh looks different. In terms of adhesion strength, SCM 180 can be attributed to the lowest adhesive strength score of 0 B (Figure 9a right part, complete removal of the Cu mesh is observed) according to ASTM D3359. At the stage of applying the notch mesh, delamination of the Cu mesh from the substrate was observed (Figure 9a left part). Moreover, the type of template does not significantly affect the adhesive strength.

Tests showed no damage to the mesh for the embedded Cu mesh, regardless of template type and plating time (Figure 9b). Thus, embedded Cu meshes e-LCM 30 and 180 and e-SCM 30 and 180 can be assigned to adhesive strength class 5B, the highest value according to ASTM D3359. Our results correlate well with the literature data for embedded metal transparent conductors based on micro and nanostructures [42,43,44].

The second approach is a cyclic tape test. It shows how the sheet resistance of a coating changes with repeated exposure. We have studied the effect of the cyclic tape test (Figure 9c) on sheet resistance of all types of coatings. Cyclic tape test data and measurements according to ASTM D3359 give similar results.

**Figure 10:**
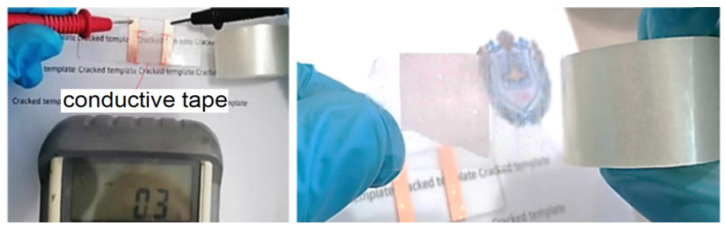
Example of obtaining electrically conductive scotch tape based on SCM 180.

Low adhesion of LCM 180 and SCM 180 enables the defect-free transfer of electroplated meshes into the adhesive layer of scotch tape [45]. This feature of electroplated Cu meshes can be an interesting practical result (Figure 10). The large non-metallized area allows the conductive tape to retain its high adhesive strength.

**Figure 11:**
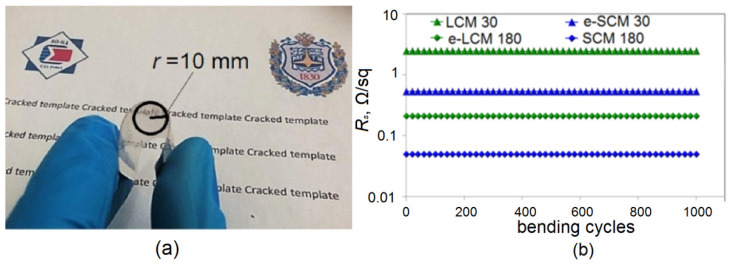
Photo e-LCM 180 in bend state (**a**); Bending stability of Cu meshes and embedded Cu meshes (**b**).

Flexural deformation resistance is also an important characteristic of coatings. In general, embedded Cu meshes are at the same level as before the embedding process. The reason for this is the high resistance of transparent coatings based on metallic micro and nanostructures to tensile–compressive deformations [31]. Figure 11a shows a graph of the effect of cyclic bending with a radius of 10 mm on sheet resistance of Cu meshes.

It can be seen that 1000 bending cycles with a radius of 10 mm do not increase sheet resistance for Cu meshes nor embedded Cu meshes.

### 3.5. Shielding Properties of Embedded Cu Meshes

The phenomenon of shielding electromagnetic radiation by a material is based on the processes of reflection, absorption and dissipation of the energy of an electromagnetic wave (EMW) in a material. A quantitative assessment of the shielding efficiency is the shielding coefficient *SE*, determined by (5) (dB).

(5) $$SE=20\mathrm{lg}\frac{E_{1}}{E_{2}}$$

where *E*_1_ is the EMW amplitude at an arbitrary point of the shielding space without the screen and *E*_2_ is the EMW amplitude at the same point with the screen. Concerning the material, the shielding coefficient can be interpreted as a value opposite in sign to the generally accepted transmission coefficient *T* (dB) (6):

(6) SE=−T

The main shielding mechanisms are absorption (*A*) and reflection (*R*); according to the law of conservation of energy, these coefficients are related by the relation *A + R + T =* 1 [46]. The absorption mechanism as the main shielding mechanism is fully characteristic of graphene [47]. Single-walled carbon nanotubes [48], conducting polymers [49] and Ag nanowires [50] have a mixed shielding mechanism (absorption and reflection in different proportions), and with a decrease in sheet resistance, the shielding mechanism shifts towards reflection. For micro and nanomesh conductive coatings with low sheet resistance, which include the copper mesh offered by us, reflection is the main shielding mechanism. More than 80–90% of all incident radiation is reflected [51]. A decrease in the specific surface resistance leads to an increase in the fraction of reflected radiation up to 99% [23].

**Figure 12:**
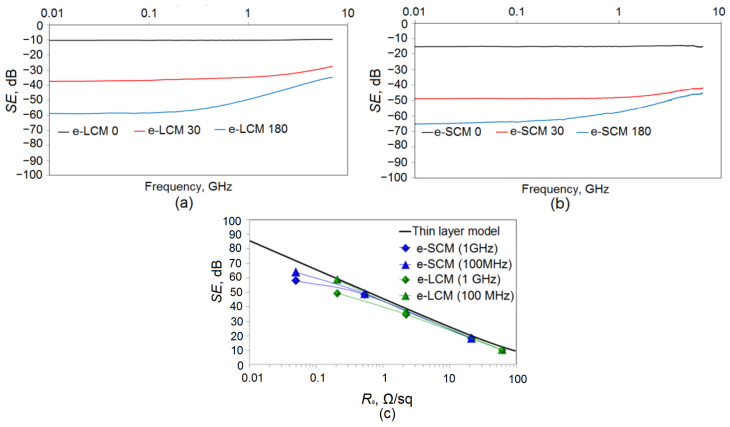
*SE* parameter for e-LCM (**a**) and e-SCM (**b**); Comparison of the *SE* parameter for e-LCM and e-SCM at frequencies of 100 MHz and 1 GHz with a continuous layer model (**c**).

Figure 12 shows the spectral dependences of the *SE* parameter for the e-LCM (Figure 12a) and e-SCM (Figure 12b).

The graphs clearly show the relationship between sheet resistance and *SE* value. Electroplating copper on Cu seed meshes leads to an intense increase in *SE*. Thus, for e-LCM (Figure 12a), an increase in the electroplating time leads to a significant increase in *SE*, from 10.17 dB to 37.41 dB (for 30 s) and 58.75 dB (180 s) at 10 MHz. For e-SCM (Figure 12b), there is a significant increase in *SE*, from 15.12 dB to 48.96 dB (for 30 s) and 65.03 dB (180 s) at 10 MHz.

Figure 12a,b shows the following: for e-LCM 0 and e-SCM 0 there is no frequency dependence in the studied range. An increase in the electroplating time leads to an intense increase in *SE*, due to a decrease in sheet resistance. In addition, the frequency dependence of *SE* becomes more pronounced. Thus, for e-LCM 30 the *SE* difference at the boundaries of the range is 9.1 dB, while for e-SCM this value is 6.44 dB. A further decrease in sheet resistance gives an even more significant difference. For the e-LCM 180 the *SE* difference is 23.26 dB; for the e-SCM 180 the *SE* difference is 18.88 dB. The average cell size makes a significant contribution to the frequency dependence of *SE*. The frequency dependence of *SE* is more pronounced for e-LCM, consistent with modern concepts of shielding electromagnetic radiation by perforated films.

According to the continuous layer model [33], which describes the relationship between sheet resistance and *SE* by the Equation (7).

(7) $$SE=20\mathrm{lg}\left(1+\frac{Z_{0}}{2R_{s}}\right)$$

where *R_s_* is the sheet resistance, *Z*_0_ = 377 Ω is the wave impedance of free space. At frequencies less than 100 MHz, agreement with the continuous layer model is quite good (Figure 12c). According to the solid layer model, a coating with a sheet resistance of 0.2 Ω/sq (such as an e-LCM 180) should have an *SE* of 59.5 dB, which we see in practice at 10 MHz. At frequencies of 1 GHz and higher, the discrepancy with the continuous layer model increases, as shown in Figure 12c. In the microwave region, even the most low-resistance coatings with sheet resistance less than 0.1 Ω/sq have an *SE* value of no more than 40–45 dB [21,23].

Coatings with a continuous structure are less susceptible to this pattern, which is confirmed in the experiment; however, these coatings rarely have a sheet resistance of less than 5 Ω/sq [33]. According to the continuous layer model, the *SE* value for such a coating should be 31.75 dB, which is in good agreement with the experimental data on the AZO/Ag/TiO_2_ structures [33].

**Figure 13:**
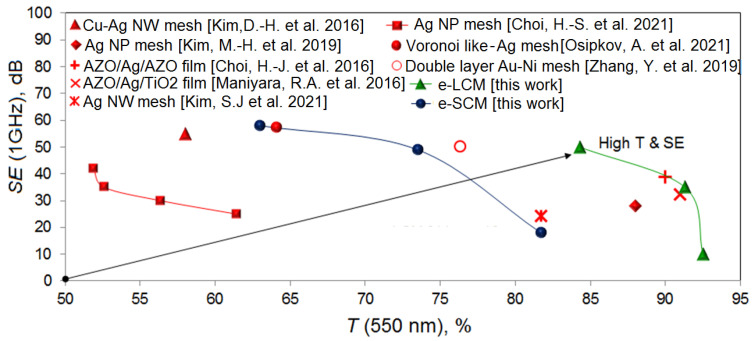
Comparative relationship between optical transparency and shielding efficiency for our and the most promising literature results [17,24,32,33,52,53,54,55].

Figure 13 shows a comparison of different types of coatings in terms of transparency and *SE* parameters at a frequency of 1 GHz.

Thus, the comparative graph shows that the e-LCM and e-SCM coatings proposed in our work correlate or surpass the most promising solutions in this direction. Thus, it can be concluded that, from the point of view of balance, the shielding efficiency-optical transparency of LCM is better than SCM. This thesis is valid only for the low-frequency range up to 1 GHz. At frequencies above 1 GHz, meshes with a large cell size demonstrate a fairly rapid drop in shielding efficiency. This is more pronounced for commercial shielding materials in which the cell size is on the order of 500 µm. Meshes with a small cell size retain high shielding efficiency over a much wider frequency range. Thus, it can be said that, in order to obtain effective coatings in a wide frequency range, it is necessary to reduce the cell size, as well as keep the crack width less than 5 μm.

## 4. Conclusions

High-quality transparent conductive coatings are made by electroplating Cu onto a thin Cu seed mesh, using the cracked template technique, followed by embedding into a photocurable resin. The resulting coatings exhibit excellent results in terms of transparent EMI shielding coatings. In particular, a coating with optical transparency of 84.3% and shielding efficiency of 49.83 dB at a frequency of 1 GHz was obtained. The maximum *SE* value for this coating was 58.75 dB at 10 MHz. In terms of the combination of optoelectric, physical and mechanical shielding properties, these coatings are among the leading coatings in modern literature.

## Figures and Tables

**Table 1:** Parameters of Cu meshes obtained using AFM.

Type Coating	Average *h*, nm	Average Error *h*, nm	Average *b*, nm	Average Error *b*, nm	*FF*, %
LCM 0	168.40	9.34	6918.72	1038.34	7.62
LCM 30	443.14	19.75	7050.13	1765.39	7.85
LCM 180	3722.82	66.35	11,573.96	1150.11	13.5
SCM 0	36.20	6.13	4251.26	396.96	11.8
SCM 30	723.03	50.15	7866.75	1088.62	19.5
SCM 180	4792.41	99.68	12,427.67	1793.26	29.9

**Table 2:** Comparison of optoelectric parameters of various transparent conductive coatings.

Type of Coating	*R_s_*, Ω/sq	T (550 nm), %	*σ_dc_/σ_opt_*	Reference
AZO/Ag/AZO	7	90	491.5	[32]
AZO/Ag/TiO_2_	5.75	91.6	730	[33]
Ag nanowires	5	92	884.9	[34]
PDDA/Ag nanowires composite	22	95.5	443	[35]
PEDOT:PSS/Ag nanowires	3	81.1	1131.1	[36]
Cu nanowires	51.5	93.1	98.2	[37]
Ag mesh (PL template)	8.2	80.2	197	[38]
AZO/Cu mesh (PL template)	6.197	90.657	605.3	[39]
Au mesh (cracked template)	3.2	92	1386.1	[40]
AgNP mesh (Emulsion template)	8.2	88	347.7	[24]
Cu mesh (Biomimic Vein-Like template)	0.1	80	15,708	[41]
e-LCM 30	2.43	91.2	1611.1	
e-LCM 180	0.21	84.3	10,189.2	This work
e-SCM 30	0.53	73.8	2179.2	
e-SCM 180	0.049	63.2	14,842.5	This work

## Data Availability

Not applicable.
